# Thyroid Function and the Risk of Non-Alcoholic Fatty Liver Disease in Morbid Obesity

**DOI:** 10.3389/fendo.2020.572128

**Published:** 2020-10-28

**Authors:** Marta Borges-Canha, João Sérgio Neves, Fernando Mendonça, Maria Manuel Silva, Cláudia Costa, Pedro M. Cabral, Vanessa Guerreiro, Rita Lourenço, Patrícia Meira, Daniela Salazar, Maria João Ferreira, Jorge Pedro, Ana Leite, Madalena Von-Hafe, Catarina Vale, Sara Viana, Ana Sande, Sandra Belo, Eva Lau, Paula Freitas, Davide Carvalho

**Affiliations:** ^1^ Serviço de Endocrinologia, Diabetes e Metabolismo do Centro Hospitalar Universitário de São João, Porto, Portugal; ^2^ Departamento de Cirurgia e Fisiologia, Faculdade de Medicina da Universidade do Porto, Porto, Portugal; ^3^ Serviço de Endocrinologia do Instituto Português de Oncologia do Porto, Porto, Portugal; ^4^ Serviço de Patologia Clínica do Centro Hospitalar, Universitário Cova da Beira, Covilhã, Portugal; ^5^ Faculdade de Ciências da Nutrição e Alimentação da Universidade do Porto, Porto, Portugal; ^6^ Investigação e Inovação em Saúde (i3s), Faculdade de Medicina da Universidade do Porto, Porto, Portugal

**Keywords:** hypothyroidism, fatty liver, non-alcoholic fatty liver disease (NAFLD), obesity, thyroid function

## Abstract

**Background:**

An association between hypothyroidism and the risk of Non-alcoholic Fatty Liver Disease (NAFLD) has been suggested. This association remains to be elucidated in patients with morbid obesity.

**Aim:**

To evaluate the association between thyroid function and parameters of liver function and hepatic scores in patients with morbid obesity.

**Methods:**

Patients with morbid obesity followed in our center between January 2010 and July 2018 were included. The ones without evaluation of liver and thyroid functions were excluded. *Fatty Liver Index* (FLI) and BARD scores were used as predictors of hepatic steatosis and fibrosis, respectively.

**Results:**

We observed a positive association between TSH and both BARD (OR 1.14; p = 0.035) and FLI (OR 1.19; p = 0.010) in the unadjusted analysis. We found a negative association between free triiodothyronine levels and BARD (OR 0.70; p<0.01) and a positive association between free triiodothyronine levels and FLI (OR 1.48; p = 0.022). Concerning liver function, we found a positive association between total bilirubin and free thyroxine levels (β = 0.18 [0.02 to 0.35]; p = 0.033) and a negative association between total bilirubin and free triiodothyronine levels (β = −0.07 [−0.14 to −0.002]; p = 0.042).

**Conclusion:**

Higher levels of TSH and free triiodothyronine may be associated with a higher risk of NAFLD, particularly steatosis, in patients with morbid obesity.

## Introduction

Non-alcoholic fatty liver disease (NAFLD) is a metabolic liver disease characterized by an extensive continuum of liver pathology, ranging from simple steatosis to steatohepatitis (NASH) and fibrosis. It can ultimately lead to cirrhosis and hepatocarcinoma (1). NAFLD comprises a massive socioeconomic burden, as it now represents the most common cause of chronic hepatic disease worldwide (2). It is becoming more prevalent and its increasing prevalence parallels the increase in metabolic syndrome and obesity. NAFLD and obesity are strongly associated and almost 80% patients with NAFLD are obese (3). Particularly, morbid obesity is believed to carry a higher risk of fibrosis and cirrhosis (3).

Even though the pathophysiology of NAFLD has been extensively studied, there is still a lot to uncover. Thyroid dysfunction, explicitly hypothyroidism, has been proposed as a possible contributory mechanism (4, 5). Indeed, it is biologically plausible for this endocrine axis to play an important role in NAFLD’s pathophysiology, as thyroid hormones (TH) are crucial in the regulation of several metabolic features such as lipid metabolism, body weight and insulin resistance (4, 6). Although many authors have been studying this hypothesis, this association in patients with morbid obesity remains to be elucidated.

In this work, we aimed to evaluate the association of thyroid function with parameters of liver function and scores predictors of hepatic steatosis and fibrosis, in patients with morbid obesity.

## Methods

### Study Design and Participants

We performed a cross-sectional study according to Strengthening the Reporting of Observational Studies in Epidemiology (STROBE) Statement (7). The study was performed in patients with morbid obesity, followed by a specialized multidisciplinary team in a specific appointment, before being submitted to bariatric surgery in our center, between January 2010 and July 2018. The data was gathered from the baseline evaluation (first appointment). Patients were excluded if they had missing liver function (n = 255) or thyroid function (n = 77). Of the 2,595 patients evaluated in our institution during the study period, 2,263 patients were included in this analysis, after applying the exclusion criteria. For this type of study formal consent is not required.

### Clinical and Biochemical Parameters Evaluated

The following parameters were evaluated: age, sex, weight, body mass index (BMI), waist and hip circumferences, history of diabetes, dyslipidemia, hypertension and treatment with levothyroxine (LT4). Diabetes was defined by fasting plasma glucose ≥126 mg/dl, glycated hemoglobin ≥6.5%, 2 h plasma glucose after a 75-g oral glucose tolerance test ≥200 mg/dl, or the use of antihyperglycemic drugs (8). Hypertension was defined as systolic blood pressure ≥140 mmHg, diastolic blood pressure ≥90 mmHg or the use of antihypertensive drugs (9). Dyslipidemia was defined by the use of lipid-lowering agents, serum low-density lipoprotein (LDL) cholesterol ≥160 mg/dl, serum high-density lipoprotein (HDL) cholesterol <40 mg/dl, or serum triglycerides ≥200 mg/dl (10). We defined as euthyroid individuals with thyroid stimulating hormone (TSH) in the reference range (0.35–4.94 μIU/ml). Albumin, triglycerides, aspartate transaminase (AST), alanine transaminase (ALT), gamma-glutamyltransferase (GGT), alkaline phosphatase (ALP), total bilirubin, direct bilirubin, TSH, free triiodothyronine (FT3), and FT4 were measured on serum, obtained from blood samples during clinical evaluations, by chemiluminescence immunoassay on the Abbott Diagnostics Architect system (Abbott Diagnostics).

### Predictors of Hepatic Fibrosis and Steatosis

As previously stated, we used the FLI and BARD scores as predictors of hepatic steatosis and fibrosis, respectively. These are built based on the following formulas:

1) FLI score: FLI = e^y^/(1+ e^y^) x 100, where y = 0.953 x ln(triglycerides, mg/dl) + 0.139 x BMI, kg/m^2^ + 0.718 x ln(GGT, U/L) + 0.053 x waist circumference, cm – 15.745. FLI scores <30 indicate low risk of hepatic steatosis, 30 to 60 intermediate risk and ≥60 high risk (11).

2) BARD score: BMI≥28 = 1 point; AST/ALT ratio≥0.8 = 2 points, presence of diabetes = 1 point. Low fibrosis risk patients are scored 0 to 1 points and higher risk patients are scored 2 to 4 points (1).

### Statistical Analysis

For continuous variables, independent t tests were performed. To evaluate the association between thyroid function and liver function parameters we used linear regression models. To evaluate the association of thyroid function with the hepatic scores, FLI and BARD, we used ordered logistic regression models. We performed the analysis unadjusted, adjusted for sex and age (model 1) and adjusted for sex, age, BMI, diabetes and dyslipidemia (model 2). We performed the main analysis with the entire population and a supplementary analysis restricted to euthyroid individuals not treated with LT4 and without past history of thyroid disease individuals or taking antithyroid drugs. Results are presented as mean ± standard deviation for continuous variables and as percentages for categorical variables. Statistical analyses were performed with Stata software, version 14.1 (StataCorp). We considered a two-sided P value less than 0.05 to be statistically significant.

## Results

### Baseline Population Characteristics

In **Table 1** we show the clinical and demographic characteristics of the population included. We included 2,263 individuals, from which 84.4% were females. The individuals were in average 42.9 ± 10.7 years old, their average weight was 115.6 ± 18.8 kg, BMI was 43.8 ± 5.7 kg/m^2^ and waist and hip circumferences were 123.0 ± 13.3 cm and 132.1 ± 11.9 cm, respectively. Thirty-two per cent of patients had diabetes, 44.8% had dyslipidemia and 62.7% had hypertension. Ninety-five percent of the included individuals were euthyroid. Nine percent were under LT4 treatment.

**Table 1:** Clinical and demographic characteristics of the population included (n = 2,263).

Age, years	42.9 ± 10.7
Feminine sex, n (%)	1,910 (84.4)
Weight, kg	115.6 ± 18.8
Body mass index, kg/m^2^	43.8 ± 5.7
Waist circumference, cm	123.0 ± 13.3
Hip circumference, cm	132.1 ± 11.9
Diabetes mellitus, n (%)	605 (32.4)
Dyslipidemia, n (%)	999 (44.8)
Hypertension, n (%)	1,173 (62.7)
Euthyroid, n (%)	2,148 (95.3)
Hyperthyroid, n (%)	42 (1.9)
Hypothyroid, n (%)	63 (2.8)
Levothyroxine treatment, n (%)	196 (8.9)
FT4, ng/dl	1.0 ± 0.2
FT3, pg/ml	3.2 ± 0.5
TSH, UI/ml	1.8 [1.3, 2.5]
Albumin, g/L	41.4 [39.6, 43.4]
AST, U/L	22.0 [18.0, 28.0]
ALT, U/L	24.0 [17.0, 35.0]
GGT, U/L	27.0 [19.0, 41.0]
ALP, U/L	75.0 [62.0, 90.0]
Total bilirubin, mg/dl	0.52 [0.41, 0.65]
Direct bilirubin, mg/dl	0.10 [0.08, 0.13]
BARD, n (%)	
1	388 (21.9)
2	254 (14.3)
3	815 (45.9)
4	317 (17.9)
FLI	97.6 [94.2, 99.2]

Values are shown as mean ± standard deviation or as median [95% confidence interval].

AST, aspartate transaminase; ALT, alanine transaminase; ALP, alkaline phosphatase; FT3, free triiodothyronine; FT4, free thyroxine; GGT, gamma-glutamyltransferase.

Concerning missing data, FT4 and FT3 were available in 95% (n = 2,138) and 42% (n = 952) of the participants. Waist circumference was available in 75.2% (n = 1,703) of the cohort. Regarding liver biochemical parameters, more than 95% of the participants had a measurement of ALT, AST, GGT and ALP; for total and direct bilirubin, data was available in about 49% of the participants.

### Association of Thyroid Function with Parameters of Liver Function

In **Table 2** we show the association of thyroid function with liver function parameters. We observed a positive correlation between total bilirubin and FT4 (β = 0.18 [0.02 to 0.35]; p = 0.033) that is maintained after adjusting for both models. Additionally, we found a negative correlation between total bilirubin and FT3 (β = –0.07 [–0.14 to –0.002]; p = 0.042), maintained after adjusting for Model 2. The results of the analysis restricted to euthyroid patients not treated with LT4 showed were similar to results in the entire population (**Supplementary Table 1**, n = 1,954).

**Table 2:** Association of thyroid function (TSH, FT4, and FT3) with parameters of liver function in the entire included population (n = 2,263).

	TSH, UI/ml		FT4, ng/dl		FT3, pg/ml	
	β	P value	β	P value	β	P value
**Albumin**, g/L						
Non-adjusted	−0.09 (−0.30, 0.13)	0.425	−0.42 (−1.51, 0.67)	0.446	−0.36 (−0.87, 0.15)	0.164
Model 1^a^	0.001 (−0.20, 0.20)	0.990	−0.36 (−1.41, 0.68)	0.494	−0.38 (−0.89, 0.13)	0.148
Model 2^b^	0.03 (−0.21, 0.27)	0.797	−0.33 (−1.52, 0.85)	0.582	−0.34 (−0.86, 0.18)	0.202
**AST**, U/L						
Non-adjusted	−0.01 (−0.03, 0.01)	0.441	0.04 (−0.07, 0.15)	0.449	0.0003 (−0.05, 0.05)	0.990
Model 1^a^	0.001 (−0.02, 0.02)	0.950	0.06 (−0.05, 0.16)	0.276	0.01 (−0.05, 0.06)	0.787
Model 2^b^	−0.004 (−0.03, 0.02)	0.777	0.05 (−0.07, 0.17)	0.435	0.01 (−0.04, 0.06)	0.734
**ALT**, U/L						
Non-adjusted	−0.02 (−0.05, 0.01)	0.256	−0.01 (−0.15, 0.14)	0.939	0.02 (−0.05, 0.09)	0.615
Model 1^a^	−0.001 (−0.03, 0.03)	0.919	0.04 (−0.10, 0.17)	0.610	0.01 (−0.05, 0.08)	0.674
Model 2^b^	−0.003 (−0.04, 0.03)	0.855	−0.05 (−0.20, 0.10)	0.527	0.01 (−0.05, 0.08)	0.660
**GGT**, U/L						
Non-adjusted	−0.001 (−0.04, 0.03)	0.970	−0.11 (−0.28, 0.06)	0.221	0.05 (−0.03, 0.13)	0.211
Model 1^a^	0.02 (−0.02, 0.50)	0.318	−0.07 (−0.23, 0.09)	0.374	0.07 (−0.01, 0.15)	0.084
Model 2^b^	0.01 (−0.03, 0.05)	0.630	−0.09 (−0.27, 0.09)	0.310	0.08 (0.005, 0.16)	**0.037**
**ALP**, U/L						
Non-adjusted	0.01 (−0.01, 0.02)	0.511	−0.02 (−0.10, 0.07)	0.699	0.004 (−0.04, 0.05)	0.858
Model 1^a^	0.01 (−0.01, 0.02)	0.444	−0.02 (−0.11, 0.06)	0.592	0.02 (−0.02, 0.07)	0.275
Model 2^b^	0.01 (−0.02, 0.03)	0.589	−0.02 (−0.12, 0.08)	0.688	0.03 (−0.01, 0.07)	0.172
**Total bilirubin**, mg/dl						
Non-adjusted	0.02 (−0.01, 0.06)	0.175	0.18 (0.02, 0.35)	**0.033**	−0.07 (−0.14, −0.002)	**0.042**
Model 1^a^	0.03 (−0.001, 0.07)	0.059	0.19 (0.03, 0.35)	**0.023**	−0.07 (−0.13, 0.002)	0.058
Model 2^b^	0.02 (−0.01, 0.06)	0.120	0.20 (0.05, 0.35)	**<0.01**	−0.08 (−0.15, −0.02)	**0.014**
**Direct bilirubin**, mg/dl						
Non-adjusted	0.03 (−0.02, 0.07)	0.217	0.21 (−0.01, 0.44)	0.063	−0.01 (−0.11, 0.09)	0.772
Model 1^a^	0.04 (−0.01, 0.08)	0.126	0.22 (−0.01, 0.44)	0.058	−0.03 (−0.13, 0.08)	0.616
Model 2^b^	0.02 (−0.02, 0.07)	0.327	0.17 (−0.06, 0.40)	0.146	−0.05 (−0.16, 0.05)	0.295

TSH was log-transformed.

^a^Adjusted to sex and age.

^b^Adjusted to sex, age, BMI, dyslipidemia, and diabetes.

AST, aspartate transaminase; ALT, alanine transaminase; ALP, alkaline phosphatase; FT3, free triiodothyronine; FT4, free thyroxine; GGT, gamma-glutamyltransferase. The significance of bolded text indicate p values less than 0.05.

### Association of Thyroid Function with FLI and BARD Scores

**Figure 1:**
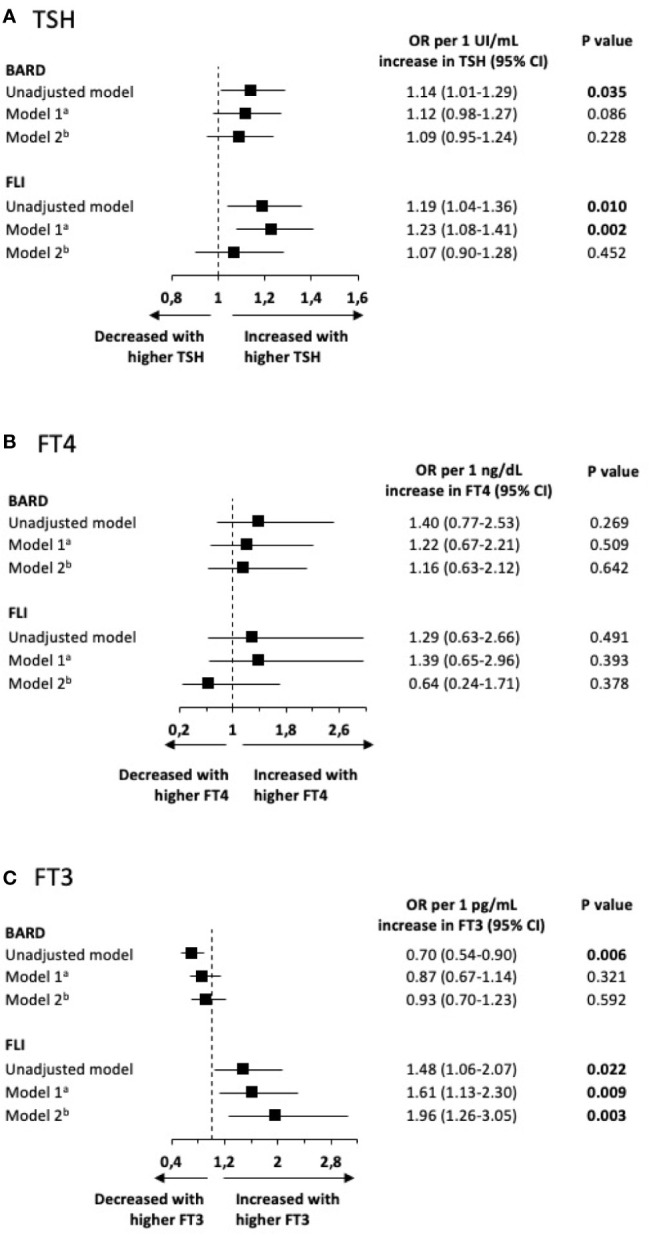
Association of thyroid function [TSH **(A)**, FT4 **(B)**, and FT3 **(C)**] with FLI and BARD scores in the entire included population (n = 2,263). **(A)** adjusted to sex and age; **(B)** adjusted to sex, age, BMI, dyslipidemia, and diabetes. FT3, free triiodothyronine; FT4, free thyroxine.

In **Figure 1**, we show the association of thyroid function with FLI and BARD scores. We observed a positive association between TSH and both FLI (OR 1.19; p = 0.011) and BARD (OR 1.14; p = 0.033) in the unadjusted analysis. In the adjusted model 1, only FLI was significantly associated with TSH. No significant associations were observed between TSH and both scores after adjusting for model 2. We also found a negative association between FT3 levels and BARD score (OR 0.70; p<0.01) and a positive association between FT3 levels and FLI score (OR 1.48; p = 0.022). Only the association between FT3 and FLI score was maintained after adjusting for both models. No association was established between FT4 and the scores tested. The results of the analysis restricted to euthyroid patients, not treated with LT4 or antithyroid drugs and without past history of thyroid disease, were analogous to those in the entire population (**Supplementary Figure 1**, n = 1,954). We also performed an analysis excluding patients with diabetes and dyslipidemia, which shows non-significant results, probably due to the metabolically healthier individuals that entered this analysis (**Supplementary Figure 2**, n = 1,050).

## Discussion

In this cross-sectional study of patients with morbid obesity, we showed a positive association of TSH and FT3 levels with the risk of hepatic steatosis as assessed by the score FLI. We also showed an association of higher levels of FT4 and lower levels of FT3 with higher levels of total bilirubin.

Although many authors have evaluated this topic, previous studies have shown contradictory results (4, 12–17). In accordance with our results, Liu et al. showed that both FT3 and TSH levels were positively correlated with the risk of NAFLD in euthyroid individuals (13). Van der Bergh et al. also showed that NAFLD patients have higher FT3 and lower FT4 levels; no differences were recorded concerning TSH (12). In a recent meta-analysis, Guo et al. concluded that TSH level may be positively correlated with NAFLD, independently of TH levels (4). On the other hand, no associations were found between thyroid function and the presence of NAFLD in a meta-analysis by Jaruvongvanich V et al (17). Of note, few previous studies evaluated the association of thyroid function with hepatic steatosis and fibrosis in patients with morbid obesity as we did in this study.

It is biologically plausible that TH have an important role in the pathogenesis of NAFLD. TH have a great impact on cholesterol and lipid metabolism, circulating lipoprotein levels and intra-hepatic lipidic concentration (18). TH regulate the expression of hepatic lipogenic genes and recent studies have shown that several genes whose expression is altered in NAFLD are also regulated by TH (19, 20). Studies in rats and humans have shown that hepatic levels of TH are decreased in NAFLD and a defective intrahepatic deiodinase expression may be a hallmark of NASH (21). Also, the literature suggests that hepatic fatty acids in NAFLD may impair TH receptors activity (22). Moreover, this apparent local hypothyroid status decreases hepatic lipases activity which promotes triglyceride accumulation (23). Furthermore, animal studies have shown that both the administration of TH and TH agonists ameliorates hepatic steatosis (24–26). The central role of TH in regulation of hepatic steatosis is further supported by recent randomized clinical trials in humans. In a double-blind, randomized, placebo-controlled trial, resmetirom, a selective thyroid hormone receptor-β agonist, lead to a significant decrease in hepatic fat content in patients with NASH, after 12 and 36 weeks of treatment (27). Bruinstroop et al. also demonstrated that low-dose TH therapy was efficacious in reducing hepatic fat content in patients with NAFLD (28).

The positive associations of both TSH and FT3 with the risk of hepatic steatosis suggest that the role of TH in the pathogenesis of NAFLD is complex. This pattern of association has also been described in obesity (and adipose tissue deposition) and in metabolic syndrome for a long time (29, 30). Hypothyroidism may contribute to some components of metabolic syndrome and the dysfunction of adipose tissue, frequently present in metabolic syndrome, may impair the homeostasis of hypothalamus-pituitary-thyroid axis (31). Previous studies have reported reductions in the expression of TH receptors in visceral and subcutaneous adipose tissue in obesity (32, 33). Additionally, studies in animal models and humans suggest that worse metabolic profiles are associated with increased type 1 deiodinase (responsible for peripheral conversion of T4 to the major active form, T3) activity in peripheral tissues (34, 35). These observations suggest that obese patients have resistance to TH which leads to a compensatory increase in TSH and FT3 levels. This is in line with our results concerning the positive association of FLI and TSH and FT3 levels. On the other hand, liver stromal cells up regulate type 3 deiodinase activity in the fibrotic liver that may culminate in a mild form of consumptive local hypothyroidism, decreasing TH hepatic levels (19, 20). This may explain the negative correlation between BARD and FT3 (although not present after the adjustments).

Finally, the positive correlation of total bilirubin with FT4 and the negative correlation with FT3 is consistent with the former hypothesis. TH stimulate the generation of bilirubin, by increasing the activity of heme oxygenase, and decrease the biliary disposal of bilirubin by inhibiting the UDP-glucuronosyltransferase activity (36, 37). The inverse association of FT3 with bilirubin levels suggests that, in this population, the higher levels of T3 may be a marker of resistance to thyroid hormones and not a marker of increased TH actions. However, there was no significant associations with AST, ALT and GGT, which are known for being stronger surrogates of NAFLD. We hypothesize that, on one hand, the association between NAFLD and TH may be far beyond those biochemical makers. On the other hand, we may have missed these results due to the population included and the cross-sectional design of the study.

There are some limitations to our work that must be acknowledged. Firstly, the cross-sectional design of this study limits our ability to evaluate the causality of associations. Furthermore, we only evaluated the parameters in a single moment, not taking into account potential variations of such parameters with acute events or offending factors (such as viral infections, that could alter both thyroid function and liver parameters). Also, other causes of hepatic disorders were not evaluated (even though we believe this limitation is strongly attenuated by the fact that NAFLD is the main cause of chronic hepatic disease worldwide (2), particularly in patients with obesity, as previously stated in our introduction). Finally, our study assessed the impact of thyroid dysfunction on NAFLD defined by non-invasive methods. We believe that these limitations are overcome by the size of the studied population and by the great importance of our results in this frequently overlooked population. Future longitudinal prospective studies using direct diagnostic methods are needed in order to corroborate our results.

In conclusion, the increase in both TSH and FT3 is associated with an increased risk of NAFLD, particularly of steatosis, as assessed by the score FLI, in patients with morbid obesity. Whether this association is influenced by confounding factors or is the result of a cause-effect pathway remains to be elucidated. There was also a negative association between FT3 and BARD, not maintained after adjustments. Our study suggests that TH may have a potential therapeutic target for the treatment of NAFLD.

## Data Availability Statement

The raw data supporting the conclusions of this article will be made available by the authors, without undue reservation.

## Ethics Statement

The studies involving human participants were reviewed and approved by C. Ética do Centro Hospitalar Universitário de São João. Written informed consent for participation was not required for this study in accordance with the national legislation and the institutional requirements.

## Author Contributions

MB-C wrote the manuscript. MB-C, JN, PF, and DC performed the statistical analysis and interpreted the results. MB-C, FM, MS, CC, PC, VG, RL, PM, DS, MF, JP, AL, MV-H, CV, SV, AS, SB, and EL gathered the data. PF, DC, and JN supervised the writing of the manuscript. All authors contributed to the article and approved the submitted version.

## Conflict of Interest

The authors declare that the research was conducted in the absence of any commercial or financial relationships that could be construed as a potential conflict of interest.

## Supplementary Material

Supplementary Figure 1 Association of thyroid function (TSH, FT4, and FT3) with FLI and BARD scores in euthyroid, non- levothyroxine supplemented individuals, without past history of thyroid disease individuals or taking antithyroid drugs (n = 1,954). ^a^ adjusted to sex and age; ^b^ adjusted to sex, age, BMI, dyslipidemia, and diabetes. FT3, free triiodothyronine; FT4, free thyroxine.

Supplementary Figure 2 Association of thyroid function (TSH, FT4, and FT3) with FLI and BARD scores excluding participants with diabetes or dyslipidemia (n = 1,050). ^a^ adjusted to sex and age; ^b^ adjusted to sex, age and BMI. FT3, free triiodothyronine; FT4, free thyroxine.

Supplementary Table 1 Association of thyroid function (TSH, FT4, and FT3) with parameters of liver function in euthyroid, non-levothyroxine supplemented, without past history of thyroid disease individuals or taking antithyroid drugs (n = 1,954). TSH was log-transformed. ^a^ Adjusted to sex and age. ^b^ Adjusted to sex, age, BMI, dyslipidemia, and diabetes. AST, aspartate transaminase; ALT, alanine transaminase; ALP, alkaline phosphatase; FT3, free triiodothyronine; FT4, free thyroxine; GGT, gamma-glutamyltransferase.
