# Supplementary figures and images for: Thyroid Function and the Risk of Non-Alcoholic Fatty Liver Disease in Morbid Obesity
Source: Front Endocrinol (Lausanne). 2020 Oct 28;11:572128. doi: 10.3389/fendo.2020.572128 (PMC7655985; doi:10.3389/fendo.2020.572128)

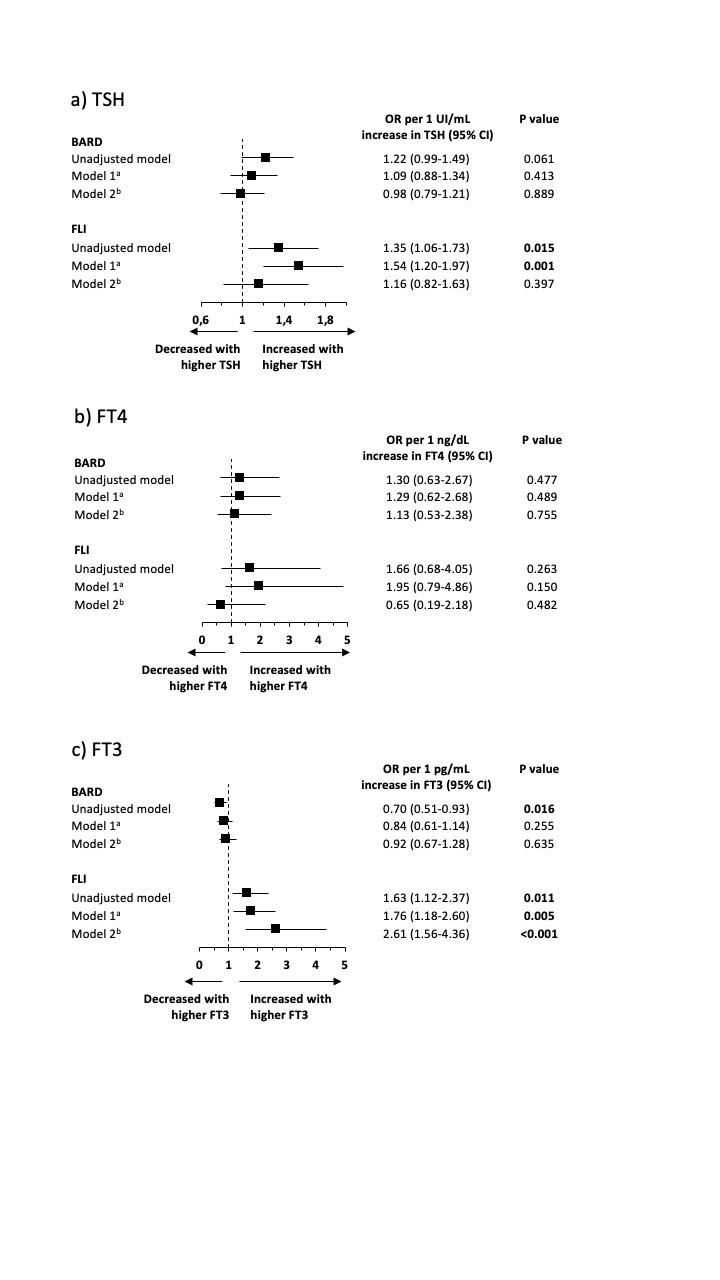

Supplement: Supplementary Figure 1 — Association of thyroid function (TSH, FT4, and FT3) with FLI and BARD scores in euthyroid, non- levothyroxine supplemented individuals, without past history of thyroid disease individuals or taking antithyroid drugs (n = 1,954). a adjusted to sex and age; b adjusted to sex, age, BMI, dyslipidemia, and diabetes. FT3, free triiodothyronine; FT4, free thyroxine. [file Image_1.jpeg]

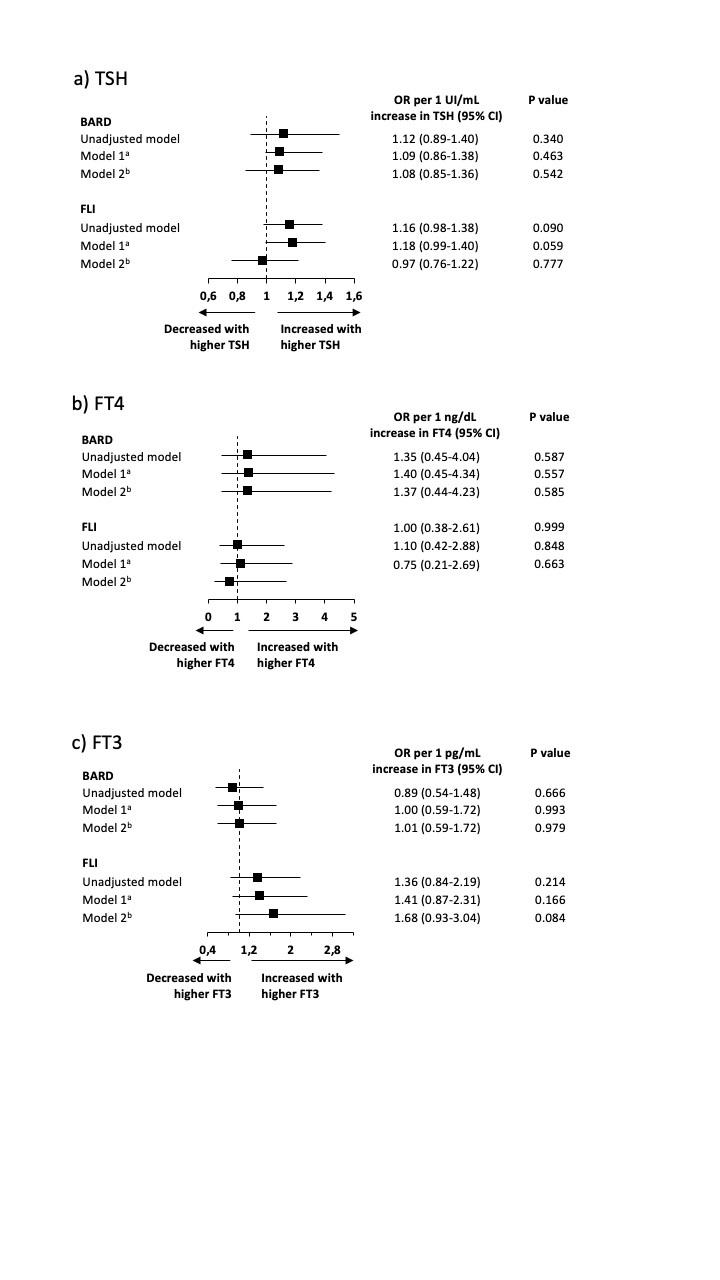

Supplement: Supplementary Figure 2 — Association of thyroid function (TSH, FT4, and FT3) with FLI and BARD scores excluding participants with diabetes or dyslipidemia (n = 1,050). a adjusted to sex and age; b adjusted to sex, age and BMI. FT3, free triiodothyronine; FT4, free thyroxine. [file Image_2.jpeg]
