# Supplementary material for: Thyroid Function and the Risk of Non-Alcoholic Fatty Liver Disease in Morbid Obesity
Source: Front Endocrinol (Lausanne). 2020 Oct 28;11:572128. doi: 10.3389/fendo.2020.572128 (PMC7655985; doi:10.3389/fendo.2020.572128)
Supplement: Supplementary Table 1 — Association of thyroid function (TSH, FT4, and FT3) with parameters of liver function in euthyroid, non-levothyroxine supplemented, without past history of thyroid disease individuals or taking antithyroid drugs (n = 1,954). TSH was log-transformed. a Adjusted to sex and age. b Adjusted to sex, age, BMI, dyslipidemia, and diabetes. AST, aspartate transaminase; ALT, alanine transaminase; ALP, alkaline phosphatase; FT3, free triiodothyronine; FT4, free thyroxine; GGT, gamma-glutamyltransferase. [file Table_1.docx]

**Tables**

| **Supplementary table 1. Association of thyroid function (TSH, FT4, and FT3) with parameters of liver function in euthyroid, non-levothyroxine supplemented, without past history of thyroid disease individuals or taking antithyroid drugs (n=1954).** | | | | | | | | | |
| --- | --- | --- | --- | --- | --- | --- | --- | --- | --- |
|  | **TSH,** UI/mL | |  | **FT4,** ng/dL | |  | **FT3**, pg/mL | | |
|  | **β** | **P value** |  | **β** | **P value** |  | **β** | **P value** |  |
| **Albumin**, g/L |  |  |  |  |  |  |  |  |  |
| Non-adjusted | -0.28 (-0.67, 0.11) | 0.159 |  | -0.58 (-1.99, 0.82) | 0.415 |  | -0.17 (-0.79, 0.44) | 0.577 |  |
| Model 1 ^a^ | -0.01 (-0.39, 0.37) | 0.952 |  | -0.43 (-1.78, 0.92) | 0.536 |  | -0.19 (-0.80, 0.43) | 0.552 |  |
| Model 2 ^b^ | 0.16 (-0.28, 0.59) | 0.479 |  | -0.57 (-2.09, 0.94) | 0.458 |  | -0.08 (-0.69, 0.53) | 0.802 |  |
| **AST**, U/L |  |  |  |  |  |  |  |  |  |
| Non-adjusted | 0.002 (-0.04, 0.04) | 0.904 |  | -0.01 (-0.15, 0.12) | 0.850 |  | 0.03 (-0.03, 0.09) | 0.348 |  |
| Model 1 ^a^ | 0.03 (-0.01, 0.06) | 0.146 |  | 0.02 (-0.11, 0.15) | 0.789 |  | 0.03 (-0.03, 0.09) | 0.297 |  |
| Model 2 ^b^ | 0.02 (-0.02, 0.06) | 0.398 |  | 0.01 (-0.14, 0.15) | 0.924 |  | 0.04 (-0.02, 0.10) | 0.207 |  |
| **ALT**, U/L |  |  |  |  |  |  |  |  |  |
| Non-adjusted | -0.01 (-0.06, 0.04) | 0.784 |  | -0.02 (-0.20, 0.17) | 0.864 |  | 0.03 (-0.04, 0.11) | 0.381 |  |
| Model 1 ^a^ | 0.04 (-0.02, 0.09) | 0.073 |  | 0.04 (-0.13, 0.21) | 0.642 |  | 0.03 (-0.04, 0.11) | 0.369 |  |
| Model 2 ^b^ | 0.03 (-0.03, 0.08) | 0.346 |  | -0.04 (-0.23, 0.14) | 0.635 |  | 0.05 (-0.02, 0.12) | 0.184 |  |
| **GGT**, U/L |  |  |  |  |  |  |  |  |  |
| Non-adjusted | 0.02 (-0.04, 0.08) | 0.468 |  | -0.11 (-0.33, 0.10) | 0.297 |  | 0.05 (-0.04, 0.14) | 0.306 |  |
| Model 1 ^a^ | 0.07 (0.02, 0.13) | **<0.01** |  | -0.06 (-0.26, 0.14) | 0.577 |  | 0.06 (-0.03, 0.15) | 0.171 |  |
| Model 2 ^b^ | 0.06 (-0.004, 0.12) | 0.068 |  | -0.04 (-0.26, 0.17) | 0.702 |  | 0.08 (-0.01, 0.17) | 0.066 |  |
| **ALP**, U/L |  |  |  |  |  |  |  |  |  |
| Non-adjusted | 0.02 (-0.01, 0.05) | 0.127 |  | -0.02 (-0.12, 0.09) | 0.753 |  | 0.02 (-0.02, 0.07) | 0.331 |  |
| Model 1 ^a^ | 0.02 (-0.03, 0.05) | 0.123 |  | -0.01 (-0.12, 0.10) | 0.874 |  | 0.04 (-0.01, 0.09) | 0.084 |  |
| Model 2 ^b^ | 0.01 (-0.02, 0.05) | 0.448 |  | 0.02 (-0.10, 0.14) | 0.775 |  | 0.05 (-0.003, 0.10) | 0.068 |  |
| **Total Bilirubin**, mg/dL |  |  |  |  |  |  |  |  |  |
| Non-adjusted | -0.004 (-0.07, 0.06) | 0.893 |  | 0.38 (0.16, 0.59) | **<0.01** |  | -0.07 (-0.14, 0.02) | 0.127 |  |
| Model 1 ^a^ | 0.02 (-0.04, 0.08) | 0.461 |  | 0.42 (0.20, 0.63) | **<0.01** |  | -0.06 (-0.14, 0.02) | 0.167 |  |
| Model 2 ^b^ | 0.01 (-0.04, 0.07) | 0.696 |  | 0.39 (0.20, 0.57) | **<0.01** |  | -0.07 (-0.15, -0.03) | **0.060** |  |
| **Direct Bilirubin**, mg/dL |  |  |  |  |  |  |  |  |  |
| Non-adjusted | -0.02 (-0.11, 0.06) | 0.571 |  | 0.34 (0.05, 0.63) | **0.023** |  | -0.03 (-0.15, 0.08) | 0.586 |  |
| Model 1 ^a^ | -0.002 (-0.08, 0.08) | 0.966 |  | 0.36 (0.07, 0.65) | **0.014** |  | -0.04 (-0.15, 0.08) | 0.551 |  |
| Model 2 ^b^ | -0.02 (-0.10, 0.07) | 0.714 |  | 0.27 (-0.03, 0.56) | 0.074 |  | -0.05 (-0.17, 0.06) | 0.372 |  |

TSH was log-transformed. ^a^ adjusted to sex and age; ^b^ adjusted to sex, age, BMI, dyslipidaemia and diabetes. AST, aspartate transaminase; ALT, alanine transaminase; ALP, alkaline phosphatase; FT3, free triiodothyronine; FT4, free thyroxine; GGT, gamma-glutamyltransferase.
